# A Mortality-Based Description of EHDV and BTV Prevalence in Farmed White-Tailed Deer (*Odocoileus virginianus*) in Florida, USA

**DOI:** 10.3390/v13081443

**Published:** 2021-07-24

**Authors:** Sydney L. Cottingham, Zoe S. White, Samantha M. Wisely, Juan M. Campos-Krauer

**Affiliations:** 1 Department of Large Animal Clinical Sciences, College of Veterinary Medicine, University of Florida, 2016 SW 16th Avenue, Gainesville, FL 32611, USA; s.cottingham@ufl.edu; 2 Department of Wildlife Ecology and Conservation, University of Florida, 110 Newins-Ziegler Hall, Gainesville, FL 32611, USA; zseganish@ufl.edu (Z.S.W.); wisely@ufl.edu (S.M.W.)

**Keywords:** epizootic hemorrhagic disease virus, EHDV, bluetongue virus, BTV, deer farming, captive cervids, *Orbivirus*

## Abstract

Hemorrhagic disease (HD) caused by bluetongue virus (BTV) and epizootic hemorrhagic disease virus (EHDV) is the most important viral disease of farmed and wild white-tailed deer (WTD; *Odocoileus virginianus*) and can cause substantial mortality in susceptible hosts. Captive cervid farming is an emerging industry in Florida, an HD-enzootic region. Morbidity and mortality due to HD are major concerns among deer farmers, but the impact of HD on Florida’s cervid farming industry is unknown. Our primary objective was to determine the prevalence of epizootic hemorrhagic disease virus (EHDV) and bluetongue virus (BTV) among WTD submitted to the University of Florida Institute of Food and Agricultural Sciences Cervidae Health Research Initiative (CHeRI) for post-mortem diagnostics. Our secondary objectives were to identify the predominant circulating EHDV serotypes during each sampling year and to determine the age class with the greatest proportion of EHDV- and BTV-positive post-mortem specimens. From 2016 to 2020, spleen samples from 539 farmed WTD with unexplained mortality were tested for the presence of EHDV and BTV by RT-qPCR. Overall, the prevalence of EHDV, BTV, or EHDV/BTV coinfection was 26%, 16%, and 10%, respectively, and 44% of deer (237/539) were diagnosed with HD by RT-qPCR. The predominant circulating EHDV serotype varied by year. Overall, EHDV-2 was the most commonly identified serotype (55% of PCR-positive cases), and EHDV-1 was the least frequently identified serotype (16% of PCR-positive cases). The greatest proportion of EHDV/BTV positives among mortality cases was observed in young WTD aged 3–6 months (50%–82% positive). There was a significant difference in the prevalence of EHDV/BTV by age when comparing specimens from WTD over 1 year old (*p* = 0.029, *n* = 527). Among these samples, the number of reported mortalities and the prevalence of EHDV/BTV were highest in yearling animals (56%). These data provide the first estimate of EHDV and BTV prevalence and virus serotypes among farmed WTD in Florida, identify the WTD age groups with the greatest proportions of EHDV- and BTV-positive specimens, and suggest that HD caused by these two viruses may be a major source of mortality challenging the captive cervid farming industry in Florida.

## 1. Introduction

Hemorrhagic disease (HD) caused by bluetongue virus (BTV) and epizootic hemorrhagic disease virus (EHDV) is the most important viral disease of farmed and wild white-tailed deer (WTD; *Odocoileus virginianus*) in North America. Although EHDV and BTV infect a wide range of domestic and wild ruminant hosts, WTD are especially susceptible to severe disease, and periodic HD outbreaks can result in significant mortality [1]. The severity of HD in wild WTD can be predicted by enzootic, epizootic, and incursive epidemiologic patterns [2]. In enzootic zones, the prevalence of seropositive individuals is high, disease incidence is low, and those individuals that do display clinical disease typically present with the chronic form of HD, characterized by mild or undetectable disease and low mortality [3,4]. In epizootic zones, periodic HD outbreaks result in higher mortality. In incursive zones, HD transmission is rare, prevalence of seropositive WTD is low, and sporadic outbreaks may produce high mortality [2,4]. Common post-mortem findings in WTD with acute HD include hyperemia of the oral mucosa and conjunctiva, pulmonary edema, pleural effusion, and multifocal hemorrhages on the serosal surfaces with multi-organ involvement [1,5].

Serotype diversity, host exposure, and subsequent acquired immunity may fluctuate spatially and temporally [6], and these factors drive disease outcomes and regional epidemiological patterns. Seven provisional EHDV serotypes (EHDV-1, -2, 4–8) and as many as 26 BTV serotypes have been detected worldwide [4]. Of these, EHDV-1 and EHDV-2 and BTV-2, -10, -11, -13, and -17 are historically endemic in the US [4]. EHDV-6 was first detected in the US in 2006 and has since become widespread [7]. In regions where multiple endemic serotypes circulate on an annual basis (e.g., southern Florida) [8,9], immunity acquired through previous infection and the passive transfer of maternal antibodies in native WTD populations may produce a state of enzootic stability [10,11,12]. In these regions, clinical disease is rare despite on-going exposure. Conversely, the risk of developing acute HD is increased in WTD that are immunologically naïve [12]. This situation is of particular relevance to the captive cervid farming industry. This industry uses controlled breeding techniques to selectively produce WTD with large antlers for high-fenced hunting preserves. Often this production goal leads to translocations of animals between farms to improve the genetic stock of a deer breeder. While importation of WTD from outside Florida is prohibited [13], movement of animals between sites within the state could lead to naïve deer being exposed after being moved to a farm with a high incidence of HD.

Due to the warm climate year-round and the presence of multiple known and potential vectors of HD in Florida, farmed cervids in the state have the potential to be exposed to EHDV and BTV beyond the “typical” HD season. EHDV and BTV are vectored by hematophagous biting midges in the genus *Culicoides* (Diptera: Ceratopogonidae), and patterns of virus transmission correspond with seasonal patterns of *Culicoides* activity and abundance. In northern latitudes, freezing temperatures limit vector activity. Consequently, most HD cases in temperate zones are reported in summer and late fall [14]. In lower latitudes where extended freezes are rare or absent, *Culicoides* may be active throughout the year, and EHDV and BTV may circulate nearly year-round [15,16]. *Culicoides sonorensis* is the only confirmed vector of EHDV and BTV in North America [17,18]. However, field-based data from insect trapping on Florida deer farms suffering HD-attributed mortality suggest *C. stellifer* and *C. venustus* as additional vectors of EHDV in Florida [19]. This study identified EHDV-positive pools of *C. venustus* and *C. stellifer* present on deer farms during an HD outbreak in North Florida, and blood meal analysis confirmed that these *Culicoides* species were feeding on farmed WTD during the peak of EHDV transmission [19]. *C. stellifer* were captured March–December, and *C. venustus* were absent only in January [19], supporting the possibility of an extended HD transmission season in Florida.

Factors associated with captivity may put farmed WTD at greater risk of infection with EHDV and BTV. A study comparing EHDV seroprevalence between farmed and wild WTD in North Florida found that farmed deer experience greater exposure to EHDV than do wild WTD, possibly due to high stocking densities or the presence of non-native ungulate species that may serve as amplifying hosts for EHDV [20]. Exotic hoofstock (Cervidae, Bovidae) are commonly stocked along with WTD on hunting preserves [21]. A bloodmeal analysis of *Culicoides* spp. on a Florida deer farm confirmed that *Culicoides* feed on exotic ungulates and suggested that the presence of these exotic ungulates may impact virus transmission to farmed WTD by changing *Culicoides* spp. abundance or feeding preferences [22]. There is no specific clinical treatment for HD aside from supportive care and treatment of secondary infections or other complications. Previously available autogenous EHDV vaccines do not produce significant antibody response in WTD [23]. An experimental bivalent vaccine for EHDV-2 and -6 is currently available nationwide to deer farmers, under special permission from the USDA. Thus, there is need both to quantify the impact of HD on the Florida deer farming industry by evaluating baseline incidence of EHDV/BTV and to identify the most frequently circulating EHDV and BTV serotypes to guide future vaccination protocols and other preventative strategies to reduce the risk of morbidity and mortality due to HD in farmed WTD with EHDV and BTV.

Here, we report data from a 5-year survey of EHDV and BTV prevalence in farmed WTD submitted for post-mortem diagnostics following unexplained death to the UF IFAS Cervidae Health Research Initiative diagnostic service (CHeRI; University of Florida, Gainesville, FL, USA). The objectives of this study were to (i) determine the prevalence of EHDV and BTV among clinical specimens submitted from WTD following unexplained death, determine the ages with the greatest proportion of EHDV- and BTV-positive cases, and (ii) identify the predominant circulating EHDV serotype by year.

## 2. Materials and Methods

### 2.1. Study Design

A determination of EHDV and BTV prevalence was implemented at the request of Florida deer farmers. Producers reported that HD due to EHDV and BTV caused significant annual mortality in breeding stock, raising concerns regarding farmed deer welfare and impact to industry profitability statewide. EHDV/BTV testing was provided through the UF IFAS CHeRI diagnostic service, free of charge to farmed deer producers in Florida. Producer participation was voluntary, and clinical samples from WTD were submitted for EHDV and BTV testing at the discretion of deer farmers based on their on-farm observation of morbidity or mortality. Only samples from dead, captive WTD from Florida deer farms were included in these analyses. Passive surveillance for EHDV/BTV in post-mortem specimens from farmed Florida WTD is ongoing, with data from August 2016 to December 2020 represented in this report.

### 2.2. Sample Collection

**Figure 1:**
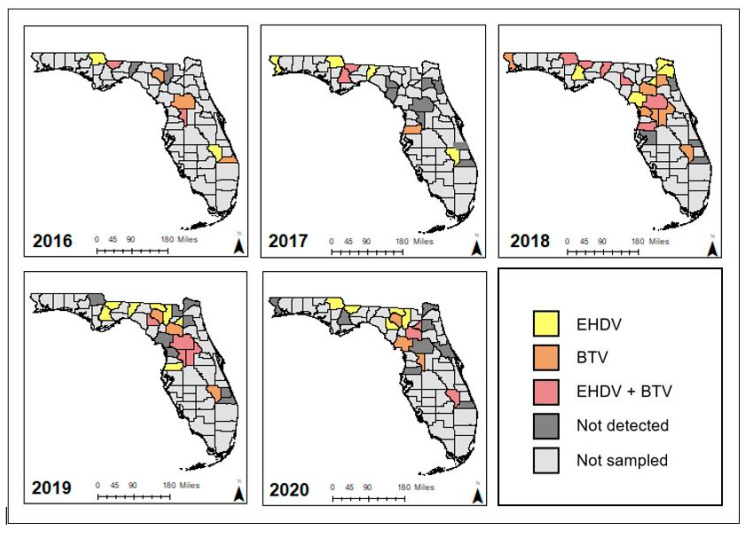
County-level sampling locations and EHDV- and BTV-positive deceased farmed Florida white-tailed deer in Florida by calendar year 2016–2020.

To determine the presence of HD-causing virus in deceased animals, spleen tissue and whole blood in ethylenediaminetetraacetic acid (BD Becton Dickinson, Franklin Lakes, NJ, USA) were collected from deceased farmed white-tailed deer submitted from Florida deer farms to the UF IFAS CHeRI diagnostic service. Diagnostic submissions were collected either by deer farm staff and shipped on cold packs or were collected on-site by CHeRI personnel, chilled, and transported directly to CHeRI laboratories. Most spleen and blood specimens were collected within 3–24 h after death, and the time between death and specimen collection did not exceed 48 h. The participation of individual deer farms and the number of submissions per farm varied by year (Figure 1). Animal signalment and clinical history were reported by deer owners, but all specimens were tested for EHDV and BTV regardless of signalment and history.

Specimen collection protocols were approved by the University of Florida Institutional Animal Care and Use Committee (IACUC protocol #201909390).

### 2.3. Laboratory Analysis

Viral RNA was extracted from EDTA whole blood or spleen tissue homogenates using the QiaAmp Viral RNA mini kit (Qiagen, Valencia, CA, USA), according to the manufacturer’s protocol. RNA was quantified using a Nanodrop One or Nanodrop 2000 (ThermoFisher, Waltham, MA, USA). Samples above 100 ng/μL were diluted to 75 ng/μL. The extracted RNA was then tested for BTV and EHDV using primers and probes described in Wernike et al. (Table 1) [24]. We carried out a 25 μL multiplex reverse transcriptase qPCR reaction (RT-qPCR) using the recommended primer and probe concentrations and VetMAX-Plus Multiplex One Step RT-PCR kit (Applied Biosystems, Foster City, CA, USA). Then, 1 μL per reaction of VetMax IPC VIC assay was added to the master mix. VetMax Xeno internal control RNA (0.1 μL or 1000 copies per reaction) was added for an internal amplification control. Molecular grade water (5.1 μL) was substituted for template RNA as a negative template control. The RT-qPCR was performed in a 7500 fast Real-Time PCR System. Cycling reaction conditions were slightly modified from the original protocol to cycle at 48 °C for 10 min, 95 °C for 10 min, 40 cycles of 95 °C for 15 sec, 57 °C for 45 s, and 68 °C for 45 sec. EHDV-2 and BTV (untyped) viral isolates collected and cultured from Florida WTD were used as positive controls. For any reaction that failed to amplify the Xeno control, the RNA was diluted 1:5 and 1:10 and then rerun. Per manufacturer’s recommendations, individual RNA samples with Ct value 38 and below were considered positive, and Ct values > 38 were considered suspect positive. The RT-qPCR protocol was repeated with suspect positive RNA samples diluted 1:5 and 1:10. The sample was determined to be positive if the Ct value was <38. If the Ct value was >38 after repeating the RT-qPCR protocol, RNA diluted samples with Ct value >38 were considered a suspect positive sample, and the result was further confirmed using virus isolation and whole genome sequencing. For EHDV-positive specimens, EHDV serotypes were differentiated and identified using a multiplex RT-PCR as described by Sun et al. [25]. PCR products were run on a 1.5% agarose gel with fluorescent stain Redview (GeneCopoeia Rockville, MD, USA) and visualized with a UVP ChemStudio Plus Imaging system (Analytik Jena, Jena, Germany).

### 2.4. Statistical Analysis

All data were analyzed in R (4.0.3) software. Summary statistics were calculated using R package “base” [26]. The number of farms and individuals sampled per farm were reported by county. The number of diagnostic samples received during the study period was reported by year. WTD specimens were analyzed by calendar year, as submissions to the CHeRI diagnostic program extended throughout the calendar year and were not limited to the typical mid-summer to late-fall HD season. Descriptive summary statistics for the annual prevalence of EHDV and BTV viral RNA were calculated separately for EHDV and BTV, and, overall, for both viruses for individual age classes. Age class was defined for juveniles in their first year of life by month up to 7 months of age (0–7+ months); yearlings and adults were defined as 1, 2, 3, or 4+ years old. We define HD as a binary variable, and samples were considered positive for EHDV or BTV when the cycle threshold (Ct) values were ≤38 cycles.

We performed Fisher’s exact test of independence with Monte Carlo simulation using the function *fisher.test* in R package “stats” to evaluate the relationship of WTD age and EHDV- and BTV-positive status [26,27]. We performed pairwise Fisher’s exact tests with Bonferroni correction for multiple comparisons using the function *pairwiseNominalIndependence* in R package “rcompanion” for post hoc analysis of significant Fisher’s exact tests [28]. We detected no significant difference in the prevalence of EHDV by age compared to the prevalence of BTV by age, and, therefore, the results of the age analysis are presented for EHDV- and BTV-positive counts combined. The number of observations for juvenile WTD aged 7–12 months was low, and there was no significant difference in EHDV/BTV prevalence in these ages, and, therefore, these observations were analyzed together (*p* > 1, *n* = 18). Likewise, the observations of adult WTD aged 4+ years were low and did not differ significantly, and these observations were analyzed together (*p* > 1, *n* = 33). Individuals for which age class data were not available were excluded from this analysis. Graphics were produced in R (4.0.3) using package “ggplot2”, and maps of the spatial distribution of sampling were produced with ArcMap GIS software (10.7.1) [29,30].

## 3. Results

### 3.1. Descriptive Sampling Summary

Statewide, specimens from 539 deer were analyzed. These specimens were sourced from 55 privately owned deer farms in 30 of 67 Florida counties over the 5-year surveillance period (Figure 1). Deer farms sampled varied by year. We were able to classify 321 WTD (165 males, 152 females, 4 unknown sex) as juveniles aged 0–12 months, and 206 WTD (97 males, 108 females, 1 unknown sex) as adults aged 13+ months. Age data were not available for 12 individuals.

### 3.2. Prevalence of EHDV and BTV in Dead Farmed Deer

Overall, 237 (44%) of 539 WTD tested positive for EHDV, BTV, or a coinfection with both viruses (Table 2). One hundred forty (26%) of these submissions were positive for EHDV, 87 (16%) tested positive for BTV, and 10 (2%) tested positive for both EHDV and BTV. EHDV prevalence was highest in 2019 (36%) and lowest in 2020 (15%). BTV prevalence was highest in 2018 (27%) and lowest in 2019 (7%). The prevalence of EHDV was higher than the prevalence of BTV in all study years. Coinfections with both EHDV and BTV were rarely detected, with 10 cases (2%) identified over the 5-year study period.

### 3.3. Predominant Circulating EHDV Serotypes

Virus serotype was determined for 81% (121/150) of WTD that tested positive for EHDV by RT-qPCR. Serotyped EHDV-positive cases are reported by year in Table 3. EHDV-1 was identified in 2019 only and was the predominant circulating serotype that year (46%). EHDV-2 was identified in every sampling year, ranging from 26%–100% of EHDV-positive cases. EHDV-2 was the only serotype identified in 2018 and 2020 and was the predominant serotype in 2016 (94%). EHDV-6 was isolated in 2016, 2017, and 2019 but not in 2018 or 2020. EHDV-6 was the predominant serotype in 2017 (71%). We identified two coinfections with EHDV-2 and EHDV-6, with one case each in 2017 and 2019. In the EHDV-positive WTD for which the EHDV serotype could not be determined, poor specimen quality due to post-mortem putrefaction or high environmental temperatures may have limited the amplification of the viral genomic sequence targeted for sequencing. BTV serotypes were not determined as part of this study.

### 3.4. Prevalence of EHDV and BTV by WTD Age

**Figure 2:**
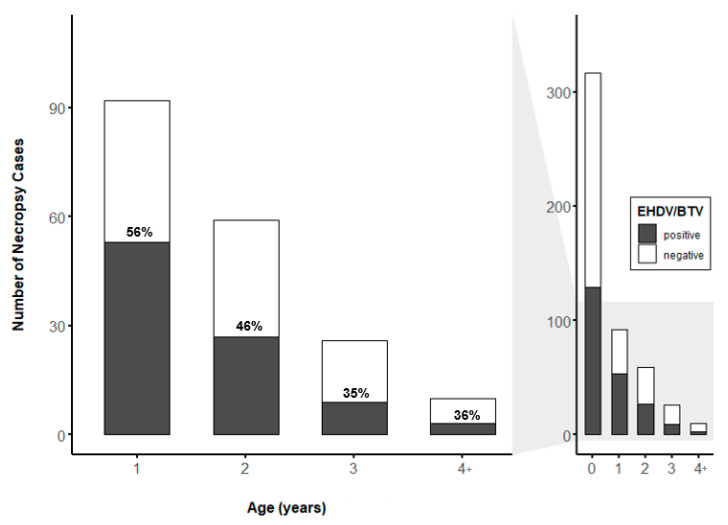
Age distribution (years) of dead farmed white-tailed deer (WTD; *n* = 527) sampled from Florida deer farms for RT-qPCR testing for epizootic hemorrhagic disease virus (EHDV) and bluetongue virus (BTV). Labels within bars represent the percentage of WTD testing positive for EHDV or BTV by age class.

Age data were available for 527 of the 539 WTD sampled, and the prevalence of EHDV and BTV were considered together. The total number of reported mortalities and EHDV/BTV prevalence varied by age class (Figure 2), though this difference was not significant when comparing the age class (yr.) of all WTD sampled (Figure 2, right) (*p* > 0.05, *n* = 527). There was a significant difference in the prevalence of EHDV/BTV by age when comparing WTD > 1 year old (Figure 2, left) (*p* = 0.029, *n* = 527). Among these samples, the number of reported mortalities and the prevalence of EHDV/BTV were highest in yearling animals (56%), but post hoc comparisons found no statistically significant difference in EHDV/BTV prevalence by age in WTD 1 year of age or older.

**Figure 3:**
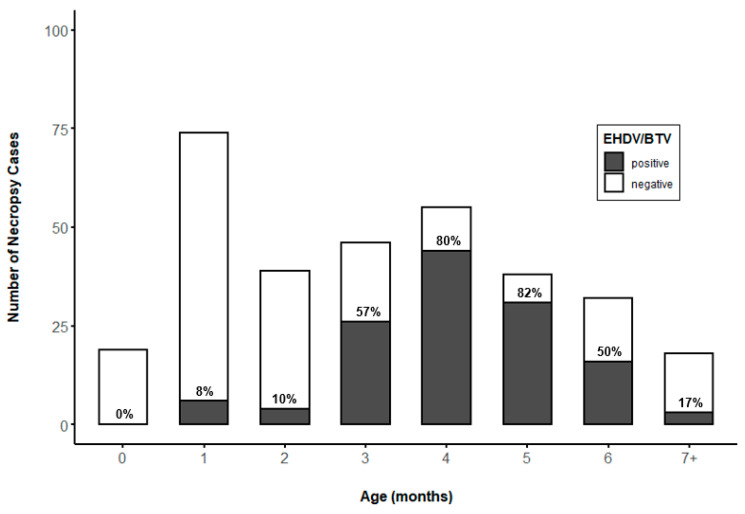
Age class distribution of dead white-tailed deer aged 0–7+ months (*n* = 321) sampled from Florida deer farms for RT-qPCR testing for epizootic hemorrhagic disease virus (EHDV) and bluetongue virus (BTV). Labels within bars represent percent of WTD testing RT-qPCR positive for EHDV or BTV by age (months).

The prevalence of EHDV/BTV varied significantly by age (mo.) in WTD younger than 1 year (*p* = 0.0005, *n* = 321). The prevalence of EHDV/BTV increased to 50%–82% in animals aged 3–6 months (Figure 3). No significant difference was found between the prevalence of EHDV/BTV in WTD aged 3–6 months, but prevalence in this age range varied significantly from EHDV/BTV prevalence observed in ages 0–2 months or 7+ months (*p* < 0.05). The number of reported mortalities was highest for 1-month-old animals, but EHDV/BTV prevalence was low (8%). The number of reported mortalities and the prevalence of EHDV/BTV declined after 7 months of age.

## 4. Discussion

Here, we report the first description of EHDV and BTV prevalence and circulating EHDV serotypes in captive WTD in Florida. Clinical specimens evaluated in this study for EHDV and BTV positivity were submitted from dead WTD. EHDV or BTV was detected in 44% of post-mortem clinical specimens from 2016–2020. While sampling dead WTD is likely to overestimate the overall prevalence of the virus, these data indicate that EHDV and BTV are associated with nearly half of the reported mortalities in farmed WTD in the state. Positive RT-qPCR test results confirmed by virus isolation and whole genome sequencing (results not presented), in addition to farmers’ on-farm observation of HD-like illness, suggest that positive RT-PCR tests are indicative of active infection with EHDV/BTV and support the use of molecular diagnostics as a high-throughput surveillance tool for EHDV and BTV in farmed WTD. The association of EHDV and BTV with 44% of reported mortalities suggests that HD due to these two viruses may pose a significant challenge to the health of farmed WTD in Florida. Full necropsy and evaluation of post-mortem findings according to a standardized case definition for HD was beyond the scope of this project. Future passive surveillance efforts should evaluate positive RT-PCR results in concert with clinical history, gross pathology, and microscopic pathology to diagnose HD in farmed deer and estimate the prevalence of HD due to EHDV and BTV in the state.

Participating farm sites varied by year, and, thus, the sampling may not fully represent HD prevalence and geographic extent over time. Notably, the sampling effort was reduced in 2020 due to institutional travel restrictions associated with the SARS-CoV-2 pandemic. Despite these limitations presented by sampling methodology, surveillance by this program is likely to identify HD outbreaks in farmed deer on participating farms and the serotypes associated with those outbreaks, as deer farmers are more likely to provide clinical submissions when herd mortality increases. Thus, the prevalence values reported here should be considered upper limit values for farmed deer populations in Florida. These prevalence estimates for EHDV and BTV provide a baseline for comparing annual patterns of EHDV and BTV exposure and mortality over time in populations of farmed deer in Florida. Additionally, these data provide a point of comparison for EHDV and BTV prevalence in farmed cervids in regions where EHDV and BTV are not enzootic.

EHDV serotype diversity among farmed WTD in Florida varied by year. All known North American EHDV serotypes (EHDV-1,2,6) were detected during the study period with EHDV-2 detected each year, 2016–2020. Circulation of a particular serotype after a period of reduced host population exposure may result in increased mortality in immunologically naïve animals. EHDV-1 was identified only in 2019 and accounted for 46% of serotyped EHDV-positive cases. Anecdotally, farmed deer herd managers reported rapid, acute death in WTD that later tested positive for EHDV-1, while producers reported a longer duration of disease in cases later associated with other EHDV serotypes by molecular diagnostics. Higher mortality rates have been reported in WTD fawns experimentally infected with EHDV-1 as compared to experimental infections with EHDV-2 [31]. EHDV-1 and EHDV-2 are considered historically endemic in North America, whereas EHDV-6 was first identified in WTD in 2006 [7,32]. EHDV-6 has since been detected in dead or moribund WTD by the Southeastern Cooperative Wildlife Disease Study (SCWDS; University of Georgia) and National Veterinary Services Laboratories (NVSL; US Department of Agriculture), every year from 2006 to 2015, including positive detections in Florida 2012–2015 [7]. These surveillance data from SCWDS and NVSL identified an outbreak of EHDV-6 in 2012 but suggest that EHDV-6 is typically present at low levels [7]. In the present study, EHDV-6 was detected in 2016, 2017, and 2019 but not in 2018 or 2020. Interestingly, EHDV-6 accounted for 71% of EHDV serotypes detected in 2017, suggesting a possible outbreak of this serotype in farmed WTD in Florida during that year. These serotype diversity data can inform vaccine development priorities by identifying the predominant EHDV serotypes associated with farmed WTD mortalities and suggest EHDV-2 as a priority for vaccine development. Primer sets used for EHDV serotyping are specific to EHDV-1, -2, and -6 and are not expected to amplify other EHDV serotypes; thus, the presence of non-endemic EHDV serotypes among the study sample cannot be excluded.

Farmed WTD in Florida may serve as sentinels for patterns of EHDV and BTV circulation in adjacent free-ranging WTD populations. A study by Cauvin et al. compared EHDV seroprevalence and titer levels in geographically related farmed and free-ranging WTD in Florida. This study found the predominant EHDV serotype to be synchronous between farmed and free-ranging populations from 2016–2017 [20]. Cauvin et al. reported EHDV-2 as the predominant serotype in 2017, as detected by a virus neutralization assay from WTD sampled March–June before the peak HD season. These data are consistent with our statewide surveillance of EHDV in farmed WTD, whereby we identified EHDV-2 as the predominant circulating serotype in 2016. The molecular diagnostics used in our study indicate the presence of viral RNA, whereas positive virus neutralization tests indicate previous exposure and subsequent antibody response. Therefore, the predominant circulation of EHDV-2 in 2016 would be reflected in EHDV-2 serotype predominance in 2017, as reported by Cauvin et al.

The probability of detection of actively circulating EHDV and BTV is higher in farmed WTD than free-ranging populations. Detection and under-reporting are inherent challenges in wildlife disease surveillance [12,33]. Typically, EHDV and BTV infections in free-ranging WTD produce subclinical or mild disease that are unlikely to be detected, and even conspicuous disease and mortality may go unreported or undiagnosed [12,33]. In contrast, the mortality of even a single WTD is highly likely to be detected by farmed deer producers during regular pen checks and routine husbandry. Furthermore, surveillance in farmed deer likely has an increased probability of detecting a virus because these specimens are from deer suffering from clinical disease or death. These disease states coincide with the peak of viremia; therefore, viral titers in blood and tissue are high and the probability of detection by high sensitivity PCR is increased [12,33,34]. Taken together, the demonstration of synchronous EHDV circulation in adjacent farmed and free-ranging WTD herds, the increased probability of detecting HD-related morbidity and mortality in farmed deer, and the increased likelihood of identifying the etiological agent of HD in these cases support farmed deer as sentinels for EHDV and BTV dynamics in free-ranging WTD. Given that the introduction of exotic strains of EHDV may impact immunologically naïve free-ranging WTD and that exotic strains of BTV present a risk both to farmed cervids and to traditional livestock industries, continued surveillance of circulating serotypes in farmed WTD is valuable to wildlife and livestock management agencies and may inform transboundary pathogen monitoring and response. Additionally, climate change may affect the latitudinal range and seasonal abundance of the *Culicoides* vector [4,35,36], and the surveillance of EHDV and BTV in farmed WTD is an additional method for monitoring changes in *Culicoides* vector ecology.

The proportion of EHDV/BTV-positive clinical submissions was highest for farmed WTD aged 3–6 months. Under typical farmed deer husbandry programs, farmed WTD are born May–July and are weaned at age 3–4 months, around September. The social and nutritional stresses of weaning are known to produce impaired immunocompetence in young ruminants, including cervids [37], and this period of high stress and immunocompromise coincides with the peak of the HD transmission season in late summer and early fall. Maternal antibodies to EHDV have been shown to reduce or prevent clinical HD in protected WTD fawns [11], but maternal antibody titers decline by around 4–5 months of age [33,38]. The concurrence of impaired immunocompetence due to weaning stress, increased infection pressure, and decay of maternal antibody titers likely explain the high proportion of EHDV/BTV-positive submissions from this age range. These data suggest that when developing EHDV vaccination programs, particular attention should be given to inducing protective immunity in young-of-year animals prior to the onset of the HD season. While vaccination at the time of weaning is convenient for producers, poor vaccine response attributed to weaning stress has been observed in farmed red deer (*Cervus elaphus*) [39]. Thus, the time of vaccine administration to high-risk young animals may be a key consideration as EHDV vaccines become commercially available for use in farmed WTD. Notably, the percentages of EHDV/BTV-positive cases among WTD aged 0–2 months were low, but the number of mortalities was high, indicating a need for further research to identify the sources of mortality in this age group.

## 5. Conclusions

These data represent the first description of EHDV and BTV prevalence and circulating EHDV serotypes in clinical submissions from deceased farmed WTD throughout Florida and provide important baseline prevalence estimates. EHDV or BTV was detected in 44% of post-mortem clinical specimens from 2016–2020 and suggests that mortality associated with EHDV and BTV may pose a significant challenge to the health, welfare, and productivity of farmed WTD in the state. EHDV-2 was identified in every sampling year and accounted for no less than one-quarter of serotyped submissions each year, suggesting this serotype as a priority for vaccine development. The proportion of EHDV/BTV-positive submissions was highest for WTD aged 3–6 months, likely due to a combination of compromised immune status during the weaning period, loss of protective maternal antibodies, and increased infection pressure during the late summer peak of seasonal EHDV and BTV transmission. Young-of-year animals should therefore be considered a priority for vaccination attempts by farmed deer producers. While EHDV and BTV were associated with slightly less than half of the deaths in farmed Florida WTD, additional work is needed to confirm HD due to EHDV/BTV as a cause of death and to determine other sources of mortality in farmed WTD. These data are essential to guide herd management, prevent future mortalities, and identify research priorities for farmed and wild WTD in Florida.

Farmed WTD may serve as indicators for EHDV and BTV dynamics in wild WTD due to the increased probability of detecting HD-related mortality in penned animals and previous work suggesting synchronous EHDV serotype dynamics in adjacent farmed and free-ranging WTD herds. The introduction of exotic strains of EHDV and BTV is a risk both to farmed cervids and to traditional livestock industries, and continued monitoring of circulating serotypes in farmed deer is valuable to transboundary pathogen monitoring and response. Continued surveillance of EHDV and BTV in farmed WTD in Florida may also provide an opportunity to detect changes is *Culicoides* ecology precipitated by anthropogenic factors, including global climate change.

## Figures and Tables

**Table 1:** Primer and probe sequences used in multiplex RT-qPCR assay for epizootic hemorrhagic disease virus (EHDV) and bluetongue virus (BTV).

Name	Sequence 5′-3′	Genomic Target	Concentration (pmol/reaction)	Reference
BTV-NS3-183F	AAA TMT TGG AYA AAG CRA TGT CAA A	NS3	10	Wernicke *et al.* [24]
BTV-NS3-288R	CTY ACR TCA TCA CGA AAC GCT		10	
BTV-NS3-242FAM	FAM-AAR GCT GCA TTC GCA TCG TAC GC-BHQ1		2	
EHD NS1 5F	AAA AAG TTC YTC GTC GAC TGC	NS1	15	Wernicke *et al.* [24]
EHD NS1 80R	ATT GGC RTA RTA ACT GTT CAT GTT		15	
EHD NS1 TAMRA	TAMRA-ATC GAG ATG GAR CGC TTY TTG AGA AAA T-BHQ2		2.5	This study

**Table 2:** Dead farmed white-tailed deer testing positive to epizootic hemorrhagic disease virus (EHDV) and bluetongue virus (BTV) by RT-qPCR from 2016–2020. Passive surveillance for EHDV/BTV in post-mortem specimens from farmed Florida WTD is ongoing.

		Positive RT-qPCR Test Results to ^a^:			
Sample Period *	*n*	EHDV Only	BTV Only	EHDV + BTV ^b^	Total Positive
2016	54	17 (31%)	12 (22%)	4 (7%)	33 (61%)
2017	118	38 (32%)	14 (12%)	1 (0.8%)	53 (45%)
2018	152	27 (18%)	42 (27%)	3 (2%)	72 (47%)
2019	126	45 (36%)	9 (7%)	2 (2%)	56 (44%)
2020	89	13 (15%)	10 (11%)	0 (0%)	23 (26%)
Total	539	140 (26%)	87 (16%)	10 (2%)	237 (44%)

* 1 January to 31 December, ^a^ Number rt-qPCR positive/number samples (% positive), ^b^ Number rt-qPCR positive to both EHDV and BTV/number samples (% positive).

**Table 3:** Epizootic hemorrhagic disease virus (EHDV) serotypes identified from dead white-tailed deer on Florida deer farms by the Cervidae Health Research Initiative (University of Florida) from 2016 to 2020.

		Serotype			
Sample Period *	*n*	EHDV-1	EHDV-2	EHDV-6	EHDV-2 + -6
2016	18	0 (0%)	17 (94%)	1 (6%)	0 (0%)
2017	31	0 (0%)	8 (26%)	22 (71%)	1 (3%)
2018	23	0 (0%)	23 (100%)	0 (0%)	0 (0%)
2019	41	19 (46%)	11 (27%)	10 (24%)	1 (2%)
2020	8	0 (0%)	8 (100%)	0 (0%)	0 (0%)
Total	121	19 (16%)	67 (55%)	33 (27%)	2 (2%)

* 1 January to 31 December, a Number rt-qPCR positive/number samples (% positive), b Number rt-qPCR positive to EHDV, BTV, or both/number samples (% positive).

## Data Availability

Not applicable.
